# Poly(ADP-Ribosyl) Code Functions

**DOI:** 10.32607/actanaturae.11089

**Published:** 2021

**Authors:** N. V. Maluchenko, D. O. Koshkina, A. V. Feofanov, V. M. Studitsky, M. P. Kirpichnikov

**Affiliations:** Lomonosov Moscow State University, Faculty of Biology, Moscow, 119234 Russia,; Shemyakin-Ovchinnikov Institute of Bioorganic Chemistry, Russian Academy of Sciences, Moscow, 117997 Russia; Fox Chase Cancer Center, Philadelphia, PA, 19111-2497 USA

**Keywords:** poly-ADP-ribose, PARP, PARG, PAR code, NAD+, phase separation

## Abstract

Poly(ADP-ribosyl)ation plays a key role in cellular metabolism. Covalent poly(ADP-ribosyl)ation affects the activity of the proteins engaged in DNA repair, chromatin structure regulation, gene expression, RNA processing, ribosome biogenesis, and protein translation. Non-covalent PAR-dependent interactions are involved in the various types of cellular response to stress and viral infection, such as inflammation, hormonal signaling, and the immune response. The review discusses how structurally different poly(ADP-ribose) (PAR) molecules composed of identical monomers can differentially participate in various cellular processes acting as the so-called “PAR code.” The article describes the ability of PAR polymers to form functional biomolecular clusters through a phase-separation in response to various signals. This phase-separation contributes to rapid spatial segregation of biochemical processes and effective recruitment of the necessary components. The cellular PAR level is tightly controlled by a network of regulatory proteins: PAR code writers, readers, and erasers. Impaired PAR metabolism is associated with the development of pathological processes causing oncological, cardiovascular, and neurodegenerative diseases. Pharmacological correction of the PAR level may represent a new approach to the treatment of various diseases.

## INTRODUCTION

**Fig. 1:**
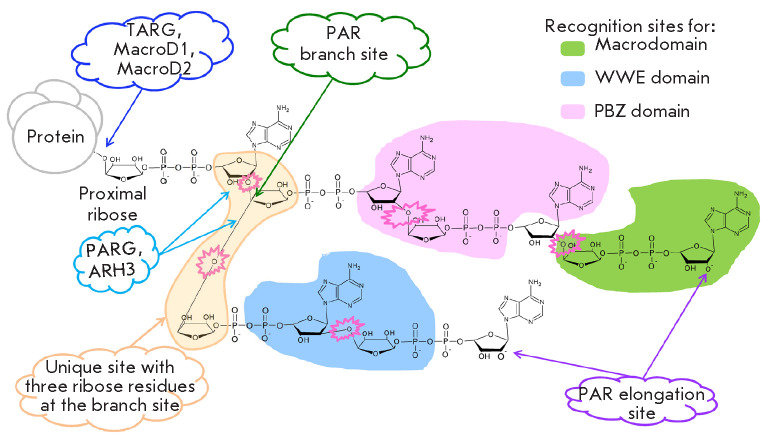
Structure of PAR on the protein globule surface and recognition sites for PAR readers and erasers. The sites for ADP-ribose elongation and branching are shown. The O-glycosidic bonds of adjacent ADP-ribose residues are encircled by a pink sawtooth line. The blue and dark blue arrows denote the sites for PAR hydrolysis by various PAR erasers. The yellow contour shows a unique site at the branch point containing three ribose residues. The areas of interaction with PAR readers containing different PAR-binding domains are denoted by green, pink, and blue contours. The green contour shows the region of the binding macrodomains that preferably interact with terminal ADP-ribose. The pink contour denotes the interaction area with PBZ domains capable of simultaneous binding to adenines in two adjacent PAR ADP-ribose units. The blue contour encircles the area of interaction with the WWE domain that recognizes iso-ADP-ribose containing a specific 2′, 1″-O-glycosidic bond

The existence of a third nucleic acid, poly(ADP-ribose) (PAR), has been known for more than half a century. Unlike DNA and RNA, PAR has a rather simple structure composed of repeating ADP-ribose (ADPR) units, but it encodes neither proteins nor RNA (*Fig. 1*) [1]. However, involvement of PAR in cell death and metabolism, as well as highly regulated synthesis, metabolism, and degradation of PAR, indicates the crucial role it plays in the cell [2, 3, 4]. Usually, PAR covalently binds to proteins and changes their activity; for this reason, poly(ADP-ribosyl)ation is often considered as a post-translational protein modification [3, 4]. This covalent modification is known to regulate the functions of the proteins involved in a number of key nuclear and cytoplasmic events, such as DNA damage repair, chromatin structure regulation, gene expression, RNA processing, ribosome biogenesis, and protein translation [4, 5, 6, 7]. In addition, there are non-covalent PAR-mediated interactions due to the presence of PAR-recognition domains in a number of proteins. Non-covalent interactions with PAR play an important role in the events determining the types of cellular response to a viral infection and stress: e.g., inflammation, hormonal signaling, and immune response [2, 8, 9, 10, 11]. A lot of evidence of PAR involvement in diseases has been accumulated. For example, β-amyloid-mediated oxidative stress in Alzheimer’s is accompanied by an increase in the PAR level; PAR also interacts with the α-synuclein that accelerates toxic fibril formation in Parkinson’s disease [12]. Numerous studies have demonstrated that there is a relationship between PAR and the processes involved in tumorigenesis [13, 14, 15, 16, 17]. As early as in 1979, poly(ADP-ribosyl) ation inhibition by nicotinamide analogs was shown to increase the sensitivity of cancer cells to cytotoxic damage [18]. To date, more than 200 similar compounds are undergoing preclinical and clinical studies as antitumor agents and four poly(ADP-ribose) polymerase (PARP) inhibitors have already been used in practice [15, 19, 20, 21, 22]. PAR is involved in cell reprogramming: intense poly(ADP-ribosyl)ation is observed in induced pluripotent stem cells, while inhibition of PAR synthesis reduces the ability of somatic cells transfected with Yamanaka factors (c-Myc, Sox2, and Oct4) to dedifferentiate [23, 24, 25]. These observations, as well as the fact that the PAR-synthesizing enzyme PARP-1 recruits the KLF4 protein to activate telomerase expression and induce stem cell pluripotency, indicate that disruptions in the PAR regulation system may lead to a more aggressive tumor stem cell phenotype. Studies on the effect of poly(ADP-ribosyl)ation on life expectancy [26, 27, 28] and progeria (Werner [29] and Cockayne [30] syndromes of premature aging) deserve special consideration. Interestingly, oxidative damage to the cell causes PARP-1 activation, which promotes cardiac and vascular dysfunction under various pathophysiological conditions [31, 32]. Pharmacological inhibition of PAR is considered a promising approach to the treatment of non-oncological diseases, such as ischemic stroke, acute pancreatitis, septic shock, asthma, and acute lung injury [19, 31, 32, 33, 34].

In general, the cellular PAR level is tightly controlled by enzymes and maintained at a low level through a finely tuned balance between the activities of poly(ADP-ribose) polymerases (PARPs) and poly(ADP-ribose) glycohydrolases (PARGs). Certain stress stimuli can rapidly increase PAR levels and trigger PAR-dependent pathways.

How can PAR molecules consisting of identical ADP-ribose monomers perform such diverse functions? How does the so-called PAR code work? This review considers the mechanisms of PAR code action, which depend on the polymer length and branching pattern, and discusses the proteins involved in code establishment, editing, and functioning.

## PROTEINS INVOLVED IN PAR SYNTHESIS

Poly(ADP-ribosyl)ation begins with PARP-mediated attachment of the first ADP-ribose moiety to an acceptor protein (usually at glutamate, aspartate, lysine, asparagine, serine, and cysteine residues). PARPs are unique glycosyltransferases that catalyze the transfer of ADP-ribosyl residues (ADPRrs) from NAD^+^ to available protein groups and subsequent chain elongation through the formation of glycosidic bonds (1’’-2’, rarely 1’’’-2’’) between the ribosyl moieties of ADP-ribose monomers. Thus, a polymer composed of two to several hundred monomers and attached covalently to the protein forms (*Fig. 1*) [35, 36, 37]. A number of chromatin- associated proteins, including core and linker histones, topoisomerases, DNA ligases, DNA polymerases, and PARPs, can act as PAR chain acceptors [5].

PAR-synthesizing proteins are often referred to as PAR writers. PARPs are the main enzymes providing the PAR structural diversity that is the basis of the PAR code. Bacterial ADP-ribosyl transferases (ADPRTs) (e.g., cholera and diphtheria toxins) and members of different yeast and animal protein families, such as arginine-specific ectoenzymes (ARTCs) and sirtuins (SIRTs), can also catalyze ADP-ribosylation.

The human PARP family includes 17 known proteins that differ in their polypeptide chain length, non-catalytic domain structure, ability to modify acceptor proteins, expression level, and intracellular distribution [2, 4, 5, 13, 38, 39]. A feature of all members of the family is a rather conserved C-terminal amino acid sequence containing a catalytic center that is a PARP signature. Most PARPs (PARP-3, 4, 6–8, 10–12, and 14–16) mono-ADP-ribosylate proteins, and only four PARPs (PARP-1, 2, 5a, and -5b), are capable of poly(ADP-ribosyl)ation. High evolutionary conservation of the primary structure of the PARP catalytic site shows that the functions of these enzymes are extremely important for the cell and the whole body. A unique feature of the PARP catalytic pocket is the ART domain, whose key motif is either the histidine- tyrosine-glutamate (HYE) triad in PARP-1–4, 5a, and 5b or the histidine-tyrosine-hydrophobic (HYφ) amino acid triad in PARP 6–8, 10–12, and 14–16 [36]. In both triads (HYE and HYφ), the conserved histidine forms a hydrogen bond with a 2-OH-ribose of the NAD^+^ adenosine, while conserved tyrosine residues form π–π stacking interactions with the NAD^+^ nicotinamide moiety. Probably, variation in the last amino acid residue in the triads controls the ability for either poly(ADP-ribosyl)ation [40] or mono-ADP-ribosylation [41]. The PARP family is currently divided into five subfamilies, based on their structural and functional features (*Table 1*).

**Table 1:** PARP subfamilies

PARP subfamilies	Subfamily members and their features
DNAdependent PARPs	DNA-dependent PARPs are activated upon DNA damage due to the presence of DNA-binding domains. The main representative, PARP-1 (ARTD1), has three DNA-binding domains (so-called zinc fingers) for damage recognition. Other subfamily members are PARP-2 (ARTD2) and PARP-3 (ARTD3).
Tankyrases	Tankyrases contain ankyrin repeats and highly specific sterile alpha motifs (SAMs) responsible for protein-protein interactions. Representatives include tankyrase-1 (PARP-5a, ARTD5) and tankyrase-2 (PARP-5b, ARTD6).
CCCH PARPs	CCCH PARPs contain a zinc finger domain with a CX_7–11_CX_3–9_CX_3_H CCCH motif interacting with RNA. These PARPs share a common WWE domain. Representatives include TIPARP (PARP-7, ARTD7), PARP-12 (ARTD12), and PARP-13 (ARTD13).
Macro PARPs	Macro PARPs contain macrodomains and mediate the association of poly- (and, possibly, mono-) ADPribosylated proteins. Representatives are BAL1 (PARP-9, ARTD9), BAL2 (PARP-14, ARTD8), and BAL3 (PARP-15, ARTD7).
Other PARPs	PARP proteins not included in the above subfamilies. Representatives are PARP-4 (ARTD4), PARP-6 (ARTD17), PARP-8 (ARTD16), PARP-10 (ARTD10), PARP-11 (ARTD11), and PARP-16 (ARTD15).

PAR synthesis is mainly performed by PARP-1 and PARP-2 (75%–95% and 5%–15%, respectively) in response to DNA damage [42, 43, 44]. Studies *in vivo* and in cell cultures have shown that a decrease in the level of PARP-1 or PARP-2 increases cell sensitivity to ionizing radiation, oxidative stress, and alkylating agents [45].

## PROTEINS HYDROLYSING PAR POLYMERS

PAR polymers are actively synthesized and hydrolyzed in the cell [6, 46]. ADP-ribosyl hydrolase 3 (ARH3), PAR glycohydrolases (PARGs), TARG/C6orf130, MacroD1, MacroD2, and NUDIX family hydrolases [2, 3, 6, 41] remove ADPR covalently bound to proteins and modulate the PAR code. All these proteins are termed PAR erasers. Many of these enzymes contain a macrodomain fold motif that allows for interaction with ADP-ribosylated substrates. PAR degradation occurs in two steps: the polymer chain is first cleaved to single ADPRrs, and the protein-bound proximal residue is then hydrolyzed (*Fig. 1*). The hydrolases PARG and ARH3 effectively cleave unique 2′–1''-glycosidic ribose–ribose bonds and release free ADPR fragments, with the proximal ADPR remaining attached to the acceptor protein [47]. Some enzymes, namely TARG, MacroD1, and MacroD2, hydrolyze an ester bond between the remaining ribose and protein acceptor amino acids, finally removing the ADPRr. The complex system of hydrolase functioning that changes the local concentration and length of PAR (i.e. modulates the PAR code) is complemented by fine regulation of specific recognition of ADPR complexed with various amino acid residues: in particular, ARH1 with Arg, ARH3 with Ser, and MacroD1, MacroD2, and TARG1 with Glu and Asp [3].

Proteins regulating PAR degradation are considered attractive therapeutic targets [6]. The first group of compounds modulating PARG activity consisted of DNA intercalators capable of association with PARs, protecting them from hydrolysis by PARGs [48]. Intercalators affect PARG activity not through direct interaction with the enzyme but by hindering its access to the substrate. Later, natural polyphenolic compounds, such as tannins directly inhibiting PARG activity, were discovered [49]. In particular, gallotannin was shown to inhibit PARG and trigger synthetic lethality in BRCA2-deficient tumors [50]. Several classes of PARG inhibitors have been studied and described so far: ADP–HPD, rhodamine inhibitors, and PDD00017273. Approaches aimed at stabilizing PARG mRNA through interaction with RNA-binding proteins (HuR) are also being developed [6, 51, 52, 53].

## PROTEINS RECOGNIZING PAR STRUCTURAL FEATURES

Proteins containing modules capable of recognizing (“reading”) the PAR structures by binding different ADPR polymer forms and acting as the so-called PAR readers have been identified over the past decade [3, 39, 54, 55, 56]. Hundreds of proteins interact with PAR directly or indirectly, thus causing subcellular redistribution of proteins and affecting many cellular processes. The structures of PAR-binding protein modules vary from highly structured domains to disordered structures (*Table 2*).

**Table 2:** PAR-recognizing modules

Module	Description	Recognition mechanism	Representatives	Functions	References
PBM	~20 a.a. [HKR] xx[AIQVY[KR]2[AILV] [FILPV] (where x stand for any amino acid)	Binding is mediated by electrostatic interactions between negatively charged PAR residues and a positively charged PBM consensus sequence; it can achieve high affinity with the complex dissociation constant (Kd) values in the submicromolar and nanomolar ranges. Interactions are enhanced by tandem arrangement of PBM modules within a protein	H1, H2A, H2B, H3, H4, p21, p53, XRCC1, XPA, MSH6, ERCC6, ATM, MRE11, DNA-PKcs, KU70, DNA ligase 3, NF-kB, TERT, DEK, CAD, CENP-A, CENP-B, lamin A/C, BUB3, hCAP-D2, HK1, HKDC1, G3BP1, hnRNPA1, hnRNPK, hnRNPH, hnRNPG, hnRNPM, iNOS hnRNPA2B1, hnRNPC1C2, AURKAIP1, RECQL5, WRN, and TOP1	PBMs are found in many proteins participating in the cellular response to DNA damage, as well as in replication, transcription, and chromatin rearrangements	[54, 55, 57, 58, 59]
Macrodomains	Evolutionarily conserved structural modules composed of ~130–190 a.a. packed into a characteristic core sandwich fold consisting of a six-stranded β-sheet surrounded by five α-helices. It is found in proteins with various cellular functions. MacroD motif: Nx(6)GG[V/L/I]D and G[V/I/A][Y/F]G	Recognition of terminal ADP-ribose residues. Kd values are in the micromolar range. ADPRbinding sites are located in the macrodomain internal cavity	Macrodomains are widespread among all kingdoms, including eukaryotes, prokaryotes, and archaea. The families are MacroH2A, MacroD, Macro2, ALC1, PARG, and SUD-M. Protein members are GDAP2, TARG1 (c6orf130), PARP-9, PARP-14, and PARP-15	Macrodomains have a regulatory effect on inter- and intracellular signaling, transcription, DNA repair, genomic stability maintenance, telomere dynamics, differentiation, proliferation, and cell death. The macrodomains of a number of proteins have catalytic activity. PARG uses a macrodomain for PAR binding and hydrolysis. MacroD and C6orf130 are involved in deacetylation of O-acetyl-ADP-ribose (a metabolite of sirtuin-mediated deacetylation of Lys). Catalytically active macrodomains in Coronaviridae, Togaviridae, and Hepeviridae viruses counteract the innate immune response, interfering with PARP-mediated antiviral protection	[60, 61, 62, 63, 64]
PBZ	~30 a.a. C2H2 type: [K/R] xxCx[F/Y] GxxCxbbxxxxHxxx[F/Y] xH	PBZ lacks secondary structure; substrate recognition is achieved through hydrogen bonds. One PBZ module is supposed to contain two binding sites that simultaneously recognize adenines in two adjacent ADPRrs in PAR, which is a distinctive feature of interaction with PBZ	APLF, CHFR, and SNM1A	DNA damage signaling. APLF promotes retention of specific NHEJ subunits in repair of double-stranded DNA breaks and stimulates the rate of NHEJ repair. CHFR is involved in regulation of the onset of mitosis	[55, 65, 66]
WWE	~80–100 a.a. Six antiparallel β-strands of the WWE domain form a half barrel structure with an α-helix in its center	Interaction occurs through phosphate groups on each iso-ADPribose side, which binds to a positively charged edge of the WWE domain. The interaction is accompanied by penetration of the adenine aromatic ring into the binding pocket. Binding is characterized by high affinity (Kd ~370 nM) and specificity	RNF146/Iduna	RNF146 is an E3 ubiquitin ligase that specifically recognizes PARconjugated protein substrates and targets them for proteasomal degradation	[67, 68]
FHA/BRCT	~80–100 a.a.	Phosphate-binding pockets interact with ADP- and iso-ADPribose residues	APTX, PNKP, XRCC1, NBS1, BARD1, and DNA ligase 4	DNA damage signaling and repair	[69]
RRM	~60–80 a.a.	The canonical RRM structure consists of four antiparallel β-strands and two α-helices located on one side of the β-sheet. The RRM domain is characterized by the presence of either 6 a.a. or 8 a.a. consensus with an exposed aromatic residue forming π–π stacking with RNA bases	Families: BRUNO, CPEB, DAZ, EIF, ELAVL, ENOX, G3BP, HNRP, IGF2BP, MSI, PABPC, PPARGC, PTBP, RALY, RAVER, RBM, RBMS, RBMY1, SAF, SF3B, SFRS, SNRP, and U2AF. Proteins: ASF/SF2, NONO, SPEN, SR140, SRRP35, SSB, SYNCRIP, TARDBP, THOC4, RBMX, TAF15, PARP- 10, and PARP-14	RNA metabolism, DNA damage signaling and repair. Targets include heterogeneous nuclear ribonucleoproteins, the proteins involved in the regulation of alternative splicing of proteins comprising small nuclear ribonucleoproteins and proteins regulating RNA stability and translation	[70, 71, 72]
SR- and KR-rich motifs	Variable	Presumably electrostatic interactions	ASF/SF2 and dMi-2	Gene expression and RNA metabolism	[54]
OB fold	~70–150 a.a. Oligonucleotide/oligosaccharide binding	Interactions with iso- ADP-ribose residues	SSB1 and BRCA2	DNA damage signaling and repair	[73]
PIN domains	~130–150 a.a.	resumably electrostatic interactions	EXO1	DNA damage signaling and repair	[74]
RG/RGG repeats	Tri-RGG: RGG(X0–4)RGG(X0–4) RGG Di-RGG RGG(X0–4) RGG Tri-RG RG(X0–4)RG(X0–4) RG Di-RG RG(X0–4) RG	Presumably electrostatic interactions. In addition, aromatic residues are often found between RGG repeats; they enable hydrophobic interactions with nitrogenous bases	Tri-RGG: FUS/TLS, EWS/EWSR1, TAF15, nucleolin, fibrillarin, SERBP1, hnRNP U, hnRNP A1, LSM14/Scd6, CHTOP, GAR1, MLL4. Di-RGG: Sam68, RPS2, hnRNP K, SYNCRIP, BRWD3, PSF, FMRP, SPRN, RasiP1 NSD1, Aven, hnRNPUL1. Tri- RG: MRE11/A, Sm-D1/ D3, KDM4E, PABP1, CIRBP, ING5, SHANK1, BAZ1A, MBD2, DDX5, DDX5, TDRD3, ILF3, 53BP1, Coilin, DHX9. Di-RG: ADAM20, E2F-1, E2F-1, Gemin 5, HMGA1, DGCR14, PDGFRB, FXR2; SRSF1, ABL2, SETD5, CPSF, BRD4, MBP, MBNL1, TGFbR, NFKBIL1, and RBBP6	Binding of various secondary RNA structures (G-quadruplexes and guanine tetrads), snRNA biogenesis, alternative splicing, translation repression (LSM14A/ Scd6), DNA damage signaling, apoptosis, G-quadruplex folding, stress granule assembly, and formation of protein condensates	[75, 76, 77, 78, 79]

## PRINCIPLES OF PAR CODE FUNCTIONING

Thus, a complex system of PAR synthesis, functioning, and degradation exists in the cell. This system regulates protein functions using the code determined by the PAR structure. The PAR code is controlled by both the PAR polymer length and the branching pattern. How does the PAR code work?

**PAR length**

**Fig. 2:**
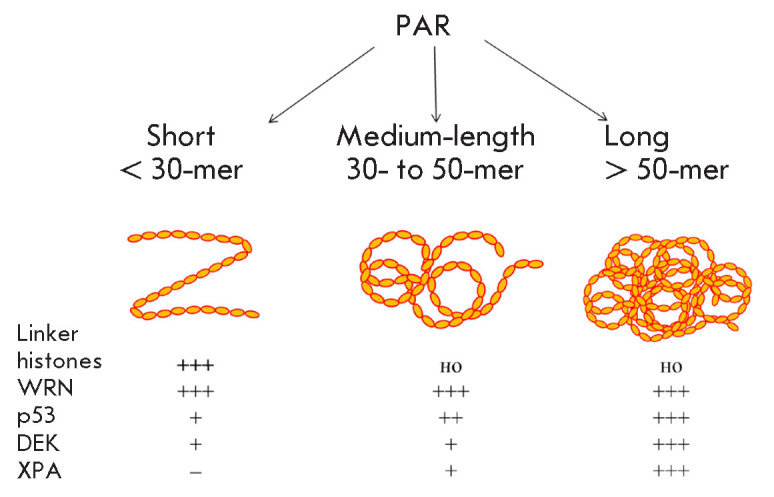
PAR length determines its association with PAR-binding proteins. The relative strength of interaction between a particular protein and PAR of a specific length is indicated by a series of crosses: “+++” –high, “++” –medium, “+” –low interaction strength, “–” –no interaction

PAR can be cytotoxic to cells under certain conditions [9]. A decrease in PARG expression, leading to PAR accumulation in the cell, enhances cell death in the presence of damaging agents both *in vitro *and *in vivo*; PARG knockout mice die on day 3.5 of embryonic development [80]. PAR-mediated cytotoxicity was previously explained by a suicide hypothesis based on cellular energy collapse caused by PARP-dependent depletion of NAD^+^ stores [81, 82]. Since the synthesis of a NAD^+^ molecule requires four ATP molecules, robust PARP activity can deplete reserves of high-energy molecules, suppress cellular energy-dependent processes such as glycolysis and mitochondrial respiration, and ultimately cause cell death [83]. However, PAR polymers themselves can be cytotoxic to cells, with the cytotoxicity level, as shown in cortical neurons, climbing with an increase in the polymer chain length and being dose-dependent (*Fig. 2*) [81]. At the same time, intracellular administration of anti-PAR antibodies significantly reduces cytotoxicity. The mechanisms of high-molecular-weight PAR cytotoxicity are being studied. The apoptosis-inducing factor (AIF) was found to be released from mitochondrial membranes in response to the treatment of isolated mitochondria with purified PARs [84]. This process also occurs in the cell’s cytoplasm, causing AIF translocation to the nucleus and cell death initiation through the mechanism of caspase-independent apoptosis. This type of programmed cell death, caused by hyperactivation of PAR synthesis, is called parthanatos. Parthanatos can be activated by severe DNA damage due to the action of alkylating agents, as well as by oxidative stress, hypoxia, hypoglycemia, and inflammation.

Depending on its length, PAR can interact with different regulatory proteins (*Fig. 2*). The human tumor suppressor protein p53 non-covalently binds to PAR and has three potential binding sites [56]. PARs longer than 50 ADPRrs are capable of high-affinity interaction with p53, while 38- to 50-mer and 5- to 38-mer PARs display moderate and weak affinity for p53, respectively [85]. Furthermore, 16- and 55-mer PARs form one and three types of complexes with the p53 protein, with dissociation constants of 250 and 130 nM, respectively [85].

Another protein interacting with PAR is the nucleotide excision repair factor XPA. XPA contains a zinc finger domain; the protein recognizes a damaged DNA region and interacts with other components of the DNA repair system. XPA does not bind to short (16-mer) PARs but forms a 1 : 1 complex with 55-mer PAR molecules (*K*d ~370 nM) [85]. A PAR-binding site overlapping with the TFIIH-recognizing region was identified in the C-terminus of the XPA protein; the TFIIH factor is involved in the initiation of transcription [86] and, together with DNA repair proteins, nucleotide excision repair [56]. It is possible that interaction with PAR may, thus, regulate XPA activity during nucleotide excision repair.

The interaction of the DEK oncoprotein with PAR also turns out to be dependent on the polymer length. DEK is involved in various intracellular processes: replication [87, 88], DNA repair [89], RNA processing [90], and transcription regulation [91, 92, 93]. High DEK levels were shown to contribute to cell immortalization, as well as suppress aging and apoptosis [94, 95]. DEK is also associated with several autoimmune disorders [96]. A number of DEK functions are regulated by either direct poly(ADP-ribosyl)ation or non-covalent interaction with PAR. Mapping of PAR-binding sites in DEK showed that the DEK region of a.a. 195–222 efficiently binds PAR, while the other two DEK regions exhibit a weaker affinity for PAR [97]. PAR chains longer than 57 ADPRrs form complexes with DEK, with a *K*d ~60 nM. PAR chains containing 34–54 ADPRrs exhibit moderate affinity for DEK; the interaction is weaker in the case of shorter polymers. Poly(ADP-ribosyl) ation disrupts the ability of DEK to bind DNA through the SAP domain, while non-covalent interactions with PAR polymers very weakly inhibit the DEK–DNA interaction [89].

Some proteins, on the contrary, efficiently interact with short PAR polymers (*Fig. 2*). For instance, histone H1 actively binds to 15- to 19-mer polymers [97]. PAR non-covalently interacts with histone H1 through the protein’s C-terminal domain, which is enriched in lysine residues [98]. Furthermore, PAR and DNA compete for binding to histone H1. PAR is suggested to be able to displace histone H1 from chromatin, preserving it in the immediate vicinity of the chain break site and, thus, implementing the “histone shuttle” mechanism [99].

We should note that the linker and core histones not only can interact non-covalently with PAR, but can also undergo covalent poly(ADP-ribosyl)ation upon PARP activation. PARP-1 and PARP-2 were shown to modify the C- and N-termini of histones H1 and H2B, respectively, causing chromatin relaxation and facilitating the recruitment of repair proteins to the damage site [100, 101, 102, 103].

The WRN factor binds equally effectively both short (10–50-mer) and long (> 50-mer) PAR polymers [104]. Interaction with PAR directly affects the WRN functions [104] that are associated with such aspects of DNA metabolism as replication, repair, and telomere length maintenance [105, 106]. A mutation in the *WRN *gene causes the hereditary Werner syndrome that is characterized by premature aging and a high risk of tumors [106], which may be explained by a high susceptibility to genotoxic stress at the cellular level. PAR can also compete with DNA for binding to the WRN N-terminal region comprising both the DNA-binding domain and the PBM domain [104]. PAR at a concentration of 10 μM inhibits WRN helicase activity, while > 50 μM PAR inhibits WRN exonuclease activity. These effects can be caused by conformational changes in WRN upon PAR binding, which lead to allosteric inhibition of the enzyme.

Why do different proteins prefer PARs of different lengths? The molecular basis for PAR recognition has not been established yet. It is possible that PAR polymers form different secondary structures, depending on their length and branching pattern (*Fig. 2*). Molecular modeling shows that five-mer PARs have a compact disordered structure, and ≥ 25-mer PARs can form several globular subdomains linked by unfolded regions [107]. As shown by circular dichroism experiments, PAR polymers (~32 units) can adopt helical conformations either in the presence of 0.1 mM spermine, 0.5 mM CaCl_2_, 0.5 mM MgCl_2_, > 3 M NaCl, or at pH > 5 [38].

**PAR branching**

**Fig. 3:**
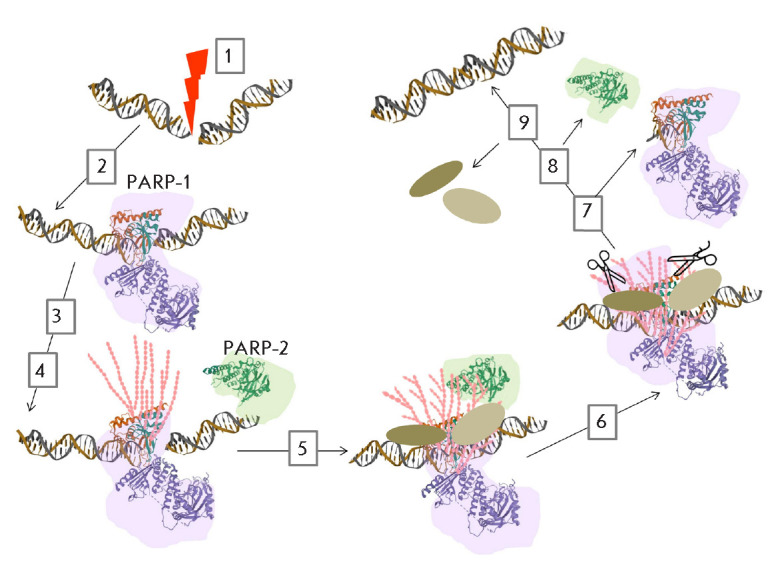
Schematic representation of the combined action of PARP-1 and PARP-2 (PAR writers) during DNA damage repair: 1) DNA damage; 2) PARP-1 is the first protein to be bound at the damage site (T1/2 ~1.6 s); 3) synthesis of primary PAR chains by PARP-1; 4) PARP-2 recruitment (after ~30 s) and accumulation (within ~2 min) at a DNA damage site; 5) synthesis of secondary PAR chains by PARP-2 and recruitment of repair factors (PAR readers); 6) degradation of PAR polymers by hydrolases (PAR erasers); 7) dissociation of PARP-1 and 8) PARP-2; 9) DNA repair and dissociation of repair factors

Although PAR branching chains were identified about 40 years ago [108], their biological functions and interactions with other cell nucleus components are still the subject of discussion. Branching PAR chains are formed with involvement of PARP-1 and PARP-2 [40, 109, 110, 111]. The unique branching pattern is achieved due to the fact that three ADP-ribose residues become linked to each other (*Fig. 1*), while known PAR-binding protein modules can recognize either one or two residues [3]. Thus, several PAR-binding domains must be coordinated to interact with the branched PAR site. Indeed, the APLF protein, which possesses two tandem PBZ domains, is capable of such binding, while the loss of the second PBZ domain switches APLF recognition from branched to linear PARs. APLF functions as a histone chaperone that preferentially binds to an H3/H4 tetramer and promotes histone release for chromatin relaxation [66, 112]. PAR chain branching provides APLF recruitment for DNA damage repair; PARP-2-deficient cells exhibit impaired kinetics of APLF recruitment to DNA damage sites. Other candidates for interaction with branched PAR sites are PARP family proteins, many of which contain tandems of PAR recognition domains [4, 38]. PARP-2 was found to interact with PAR via its N-terminal region, the so-called NTR, which lacks any specific structure [43, 113]. The PARP-2 NTR shares homology with the SAP domains of other proteins involved in chromatin organization and DNA repair, such as Ku70 and APE1 [44, 113, 114]. NTR deletion disrupted the PARP-2 ability to interact with PAR and suppressed its enzymatic activity in [109]. Since PARP-2 binds to PAR, the question arises as to whether this binding plays a significant role in the recruitment of PARP-2 to a damage site in the cell. Summarizing the data from various laboratories, we may suggest the following mechanism: PARP-1 is the first (T1/2 ~1.6 s) to occur at the damage site [7, 110, 111, 115, 116, 117, 118] and to synthesize the first PAR chains (*Fig. 3*). PARP-2 binds later (after ~30 s), accumulates at the DNA damage site (~2 min), and synthesizes secondary, predominantly branched PARs [109]. Treatment of cells with olaparib (PARP inhibitor) inhibits PARP-2 recruitment, while PARP-2 recruitment to the damage site in PARP-1-deficient cells occurs with a low efficiency [42]. These results suggest that PARP-2 recognizes PAR synthesized by PARP-1; PAR, in turn, mediates PARP-2 recruitment to the DNA damage site. In addition, PARP-1 and PARP-2 are characterized by short-term and long-term accumulation at the damage site, respectively [118].

It is also possible that branched PAR functions include recruitment of unique proteins and creation of the high-molecular-weight condensates involved in certain intracellular processes.

**PAR participation in the formation of subcellular liquid-phase structures**

**Fig. 4:**
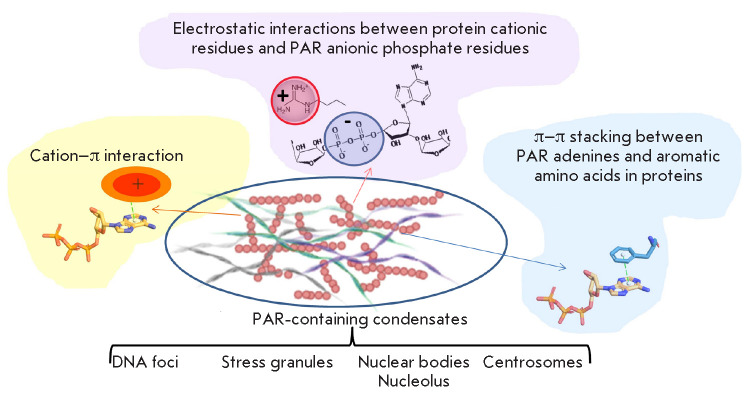
PAR-dependent biomolecular condensates formed by phase separation; the PAR–protein interactions stabilizing these condensates

Many subcellular compartments lack membranes. They form by separation of liquid phases and enable the cell to spatially separate different biochemical processe [119, 120]. Membraneless organelles (biomolecular condensates) resulting from phase transitions of macromolecular complexes include the nucleolus, nuclear bodies, Cajal bodies, DNA foci, PML bodies, and stress granules. Polymers composed of nucleic acids and proteins and containing disordered domains or, as they are usually called, low-complexity domains, play the most important role in the formation of these condensates. These domains are characterized by a tendency towards energetically favorable condensation due to weak but multivalent interactions between polymers [110, 121, 122, 123]. Single-stranded nucleic acids represent an ideal multivalent scaffold for the formation of numerous bonds with disordered protein domains and the production of biomolecular condensates [124, 125]. Currently, there is growing evidence of the important role of PAR in the initiation of the formation of these condensates (*Fig. 4*) [3]. PAR has a rather simple structure composed of repeating monomers, with a large binding surface area recognized by various proteins. PAR adenine bases occur in the anti-conformation, which exposes them to potential interaction with other molecules [126]. Furthermore, PAR is characterized by active synthesis and degradation kinetics, which allows PAR to serve as a temporary scaffold for both initiation of molecular condensates and destruction of these structures, which provides fast phase transitions “on demand,” i.e. in response to changes in the microenvironment. A number of researchers have shown that PAR induces regulated formation of molecular condensates by recruiting proteins containing disordered domains [38, 59, 127, 128]. It is possible that the PAR length, branching pattern, and concentration affect the formation of these molecular condensates through a change in the scaffold area accessible to protein binding. The electrostatic interaction between PAR and proteins, which is crucial for phase separation, can be disturbed by introducing a negative charge into the proteins (e.g., through their regulatory phosphorylation) [75].

PAR is involved in the organization of liquid-phase membraneless organelles, such as the nucleolus, stress granules, and DNA foci (DNA damage sites) [3, 38, 129]. A mechanism for the formation of membraneless repair compartments, which is mediated by interaction of disordered FUS domains with PAR, has been proposed [127]. These compartments provide highly effective repair thanks to local accumulation of repair proteins and separation of damaged DNA from intact DNA [75, 127, 130].

Other liquid-phase membraneless compartments associated with PAR are ribonucleoprotein structures: stress granules and P-bodies (*Fig. 4*). These structures are involved in RNA metabolism, including control of mRNA stability and translation [131]. Poly(ADP-ribosyl) ation serves as an important regulator of the dynamics of ribonucleoprotein complexes. Formation of ribonucleoprotein complexes during prolonged stress and excessive activation of PAR synthesis becomes pathological and leads to the formation of insoluble aggregates.

The PAR-mediated mechanism of phase transition provides for the formation of transient transcriptional complexes at expressed genes through the C-terminal domain (CTD) of RNA polymerase II, which contains a disordered domain capable of multivalent interaction [132, 133, 134]. CTD phosphorylation releases RNA polymerase II from these transcriptional complexes. PARP-1 is found at the promoters of actively transcribed genes, its activity stimulating post-translational modifications, promoting transcription; PARP-1 also displaces histone H1, thereby increasing the accessibility of DNA promoters [135, 136]. Thus, formation of transient condensates of transcriptional complexes promotes local formation of an active transcriptional environment.

## CONCLUSION

Synthesis of PARs, namely nucleic acid-like polymeric structures of varying lengths, is one of the mechanisms of adaptation and initiation of the necessary cellular processes in response to various stress stimuli. Despite the fact that, unlike DNA and RNA, the PAR sequence does not encode any information, the length and structure of PAR polymers determine the PAR code. This code is recognized by a variety of the proteins involved in repair, transcription, and organization of the chromatin structure. The cellular PAR level is inconstant; it is strictly controlled by enzymes that synthesize, recognize, and hydrolyze PARs. Liquid-phase biomolecular compartments, in which PAR acts as a scaffold for the condensation of proteins containing disordered domains, and their partners, are assembled to increase the effectiveness of certain biochemical processes: e.g., transcription, repair, and RNA biogenesis. These complexes are quickly disassembled after PAR hydrolysis. Impaired PAR metabolism is associated with the development of pathological processes, leading to oncological, cardiovascular, and neurodegenerative diseases, as well as premature aging. Therefore, PAR code-modulating proteins are considered important therapeutic targets. Indeed, several PARP inhibitors are already successfully used as anticancer agents, while others are being developed and tested. PAR-hydrolyzing enzymes are another promising target. What is more, compounds capable of controlling the PAR level may be used in the therapy of non-oncological diseases.

## Abbreviations

ADPR: ADP-ribose
ADPRr: ADP-ribose residue
PAR: poly(ADP-ribose)
PARG: poly(ADP-ribose) glycohydrolase
PARP: poly(ADP-ribose) polymerase.

## References

[1] Chambon P., Weill J.D., Mandel P. (1963). Biochem. Biophys. Res. Commun..

[2] Gupte R., Liu Z., Kraus W.L. (2017). Genes Dev..

[3] Luscher B., Butepage M., Eckei L., Krieg S., Verheugd P., Shilton B.H. (2018). Chem. Rev..

[4] Kraus W.L. (2020). Genes Dev..

[5] Sutcu H.H., Matta E., Ishchenko A.A. (2020). J. Mol. Biol..

[6] O’Sullivan J., Tedim Ferreira M., Gagne J.P., Sharma A.K., Hendzel M.J., Masson J.Y., Poirier G.G. (2019). Nat. Commun..

[7] Liu C., Vyas A., Kassab M.A., Singh A.K., Yu X. (2017). Nucleic Acids Research.

[8] Kim M.Y., Zhang T., Kraus W.L. (2005). Genes Dev..

[9] Luo X., Kraus W.L. (2012). Genes Dev..

[10] Vyas S., Chang P. (2014). Nat. Rev. Cancer..

[11] Kashima L., Idogawa M., Mita H., Shitashige M., Yamada T., Ogi K., Suzuki H., Toyota M., Ariga H., Sasaki Y. (2012). J. Biol. Chem..

[12] Kim S., Kwon S.H., Kam T.I., Panicker N., Karuppagounder S.S., Lee S., Lee J.H., Kim W.R., Kook M., Foss C.A. (2019). Neuron..

[13] Marti J.M., Fernandez-Cortes M., Serrano-Saenz S., Zamudio-Martinez E., Delgado-Bellido D., Garcia-Diaz A., Oliver F.J. (2020). Cancers (Basel)..

[14] Grimaldi G., Catara G., Palazzo L., Corteggio A., Valente C., Corda D. (2019). Biochem. Pharmacol..

[15] Nilov D., Maluchenko N., Kurgina T., Pushkarev S., Lys A., Kutuzov M., Gerasimova N., Feofanov A., Svedas V., Lavrik O., Studitsky V.M. (2020). Int. J. Mol. Sci..

[16] Malyuchenko N.V., Kotova E.Y., Kulaeva O.I., Kirpichnikov M.P., Studitskiy V.M. (2015). Acta Naturae..

[17] Masutani M., Fujimori H. (2013). Mol. Aspects Med..

[18] Terada M., Fujiki H., Marks P.A., Sugimura T. (1979). Proc. Natl. Acad. Sci. USA..

[19] Min A., Im S.A. (2020). Cancers (Basel)..

[20] Patel M., Nowsheen S., Maraboyina S., Xia F. (2020). Cell Biosci..

[21] Nilov D.K., Tararov V.I., Kulikov A.V., Zakharenko A.L., Gushchina I.V., Mikhailov S.N., Lavrik O.I., Švedas V.K. (2016). Acta Naturae..

[22] Sherstyuk Y.V., Ivanisenko N.V., Zakharenko A.L., Sukhanova M.V., Peshkov R.Y., Eltsov I.V., Kutuzov M.M., Kurgina T.A., Belousova E.A., Ivanisenko V.A. (2019). Int. J. Mol. Sci..

[23] Liu Z K.W. (2017). Molecular Cell.

[24] Yoo Y.D., Huang C.T., Zhang X., Lavaute T.M., Zhang S.C. (2011). Stem Cells..

[25] Chiou S.H., Jiang B.H., Yu Y.L., Chou S.J., Tsai P.H., Chang W.C., Chen L.K., Chen L.H., Chien Y., Chiou G.Y. (2013). J. Exp. Med..

[26] Shilovsky G.A., Shram S.I., Morgunova G.V., Khokhlov A.N. (2017). Biochemistry (Moscow)..

[27] Burkle A., Diefenbach J., Brabeck C., Beneke S. (2005). Pharmacol. Res..

[28] Muiras M.L., Muller M., Schachter F., Burkle A. (1998). J. Mol. Med. (Berlin)..

[29] Deschenes F., Massip L., Garand C., Lebel M. (2005). Human Molecular Genetics.

[30] Thorslund T., von Kobbe C., Harrigan J.A., Indig F.E., Christiansen M., Stevnsner T., Bohr V.A. (2005). Mol. Cell. Biol..

[31] Henning R.J., Bourgeois M., Harbison R.D. (2018). Cardiovasc. Toxicol..

[32] Cao S., Sun Y., Wang W., Wang B., Zhang Q., Pan C., Yuan Q., Xu F., Wei S., Chen Y. (2019). J. Cell. Mol. Med..

[33] Rao P.D., Sankrityayan H., Srivastava A., Kulkarni Y.A., Mulay S.R., Gaikwad A.B. (2020). Drug Discov. Today..

[34] Berger N.A., Besson V.C., Boulares A.H., Burkle A., Chiarugi A., Clark R.S., Curtin N.J., Cuzzocrea S., Dawson T.M., Dawson V.L. (2018). B. J. Pharmacol..

[35] D’Amours D., Desnoyers S., D’Silva I., Poirier G.G. (1999). Biochem. J..

[36] Hottiger M.O., Hassa P.O., Luscher B., Schuler H., Koch-Nolte F. (2010). Trends Biochem. Sci..

[37] Burkle A. (2005). FEBS J..

[38] Leung A.K.L. (2020). Trends Cell Biol..

[39] Karlberg T., Langelier M.F., Pascal J.M., Schuler H. (2013). Mol. Aspects Med..

[40] Rolli V., O’Farrell M., Menissier de Murcia J., de Murcia G. (1997). Biochemistry..

[41] Vyas S., Matic I., Uchima L., Rood J., Zaja R., Hay R.T., Ahel I., Chang P. (2014). Nat. Commun..

[42] Shieh W.M., Ame J.C., Wilson M.V., Wang Z.Q., Koh D.W., Jacobson M.K., Jacobson E.L. (1998). J. Biol. Chem..

[43] Ame J.C., Rolli V., Schreiber V., Niedergang C., Apiou F., Decker P., Höger T., de Murcia J.M., de Murcia G. (1999). J. Biol. Chem..

[44] Huber A., Bai P., de Murcia J.M., de Murcia G. (2004). DNA Repair (Amst.)..

[45] Menissier de Murcia J., Ricoul M., Tartier L., Niedergang C., Huber A., Dantzer F., Schreiber V., Amé J.C., Dierich A., LeMeur M. (2003). EMBO J..

[46] Tulin A.S., Spradling A. (2003). Science..

[47] Slade D.D.M., Barkauskaite E., Weston R., Lafite P., Dixon N., Ahel M., Leys D., Ahel I. (2011). Nature.

[48] Tavassoli M., Tavassoli M.H., Shall S. (1985). Biochim. Biophys. Acta..

[49] Tsai Y.J., Abe H., Maruta H., Hatano T., Nishina H., Sakagami H., Okuda T., Tanuma S. (1991). Biochem. Int..

[50] Fathers C., Drayton R.M., Solovieva S., Bryant H.E. (2012). Cell Cycle..

[51] Gravells P., Neale J., Grant E., Nathubhai A., Smith K.M., James D.I., Bryant H.E. (2018). DNA Repair (Amst.)..

[52] Chand S.N., Zarei M., Schiewer M.J., Kamath A.R., Romeo C., Lal S., Cozzitorto J.A., Nevler A., Scolaro L., Londin E. (2017). Cancer Research.

[53] Slama J.T., Aboul-Ela N., Jacobson M.K. (1995). J. Med. Chem..

[54] Teloni F., Altmeyer M. (2016). Nucleic Acids Research.

[55] Krietsch J., Rouleau M., Pic E., Ethier C., Dawson T.M., Dawson V.L., Masson J.Y., Poirier G.G., Gagné J.P. (2013). Mol. Aspects Med..

[56] Pleschke J.M.K., Kleczkowska H.E., Strohm M., Althaus F.R. (2000). J. Biol. Chem..

[57] Zhou Z.D., Chan C.H., Xiao Z.C., Tan E.K. (2011). Cell Adh. Migr..

[58] Kamaletdinova T., Fanaei-Kahrani Z., Wang Z.Q. (2019). Cells..

[59] Duan Y., Du A., Gu J., Duan G., Wang C., Gui X., Ma Z., Qian B., Deng X., Zhang K. (2019). Cell Res..

[60] Žaja R., Mikoč M.A., Barkauskaite E., Ahel I. (2013). Biomolecules..

[61] Chen D.V., Vollmar M., Rossi M.N., Phillips C., Kraehenbuehl R., Slade D., Mehrotra P.V., von Delft F., Crosthwaite S.K., Gileadi O., Denu J.M., Ahel I. (2011). J. Biol. Chem..

[62] Alhammad Y.M.O., Fehr A.R. (2020). Viruses..

[63] Grunewald M.E., Chen Y., Kuny C., Maejima T., Lease R., Ferraris D., Aikawa M., Sullivan C.S., Perlman S., Fehr A.R. (2019). PLoS Pathog..

[64] Rack J.G., Perina D., Ahel I. (2016). Annu. Rev. Biochem..

[65] Ahel I., Ahel D., Matsusaka T., Clark A.J., Pines J., Boulton S.J., West S.C. (2008). Nature.

[66] Li G.Y., McCulloch R.D., Fenton A.L., Cheung M., Meng L., Ikura M., Koch C.A. (2010). Proc. Natl. Acad. Sci. USA..

[67] Wang Z.M., Michaud G.A., Cheng Z., Zhang Y., Hinds T.R., Fan E., Cong F., Xu W. (2012). Genes Dev..

[68] Kang H.C.L., Lee Y.I., Shin J.H., Andrabi S.A., Chi Z., Gagne J.P., Lee Y., Ko H.S., Lee B.D., Poirier G.G., Dawson V.L., Dawson T.M. (2011). Proc. Natl. Acad. Sci. USA..

[69] Li M., Lu L.Y., Yang C.Y., Wang S., Yu X. (2013). Genes Dev..

[70] Vyas S., Chesarone-Cataldo M., Todorova T., Huang Y.H., Chang P. (2013). Nat. Commun..

[71] Bock F.J., Todorova T.T., Chang P. (2015). Molecular Cell.

[72] Messias A.C., Sattler M. (2004). Acc. Chem. Res..

[73] Zhang F., Chen Y., Li M., Yu X. (2014). Proc. Natl. Acad. Sci. USA..

[74] Zhang F., Shi J., Chen S.H., Bian C., Yu X. (2015). Nucleic Acids Research.

[75] Altmeyer M., Neelsen K.J., Teloni F., Pozdnyakova I., Pellegrino S., Grofte M., Rask M.B.D., Streicher W., Jungmichel S., Nielsen M.L. (2015). Nat. Commun..

[76] Chong P.A., Vernon R.M., Forman-Kay J.D. (2018). J. Mol. Biol..

[77] Thandapani P., O’Connor T.R., Bailey T.L., Richard S. (2013). Molecular Cell.

[78] Masuzawa T., Oyoshi T. (2020). ACS Omega..

[79] Ozdilek B.A., Thompson V.F., Ahmed N.S., White C.I., Batey R.T., Schwartz J.C. (2017). Nucleic Acids Research.

[80] Koh D.W., Lawler A.M., Poitras M.F., Sasaki M., Wattler S., Nehls M.C., Stöger T., Poirier G.G., Dawson V.L., Dawson T.D. (2004). Proc. Natl. Acad. Sci. USA..

[81] Andrabi S.A., Umanah G.K., Chang C., Stevens D.A., Karuppagounder S.S., Gagne J.P., Poirier G.G., Dawson V.L., Dawson T.D. (2014). Proc. Natl. Acad. Sci. USA..

[82] Berger N.A., Sims J.L., Catino D.M., Berger S.J. (1983). Princess Takamatsu Symp..

[83] Chiarugi A. (2002). Trends Pharmacol. Sci..

[84] Yu S.W., Andrabi S.A., Wang H., Kim N.S., Poirier G.G., Dawson T.M., Dawson T.D. (2006). Proc. Natl. Acad. Sci. USA..

[85] Fahrer J., Kranaster R., Altmeyer M., Marx A., Burkle A. (2007). Nucleic Acids Research.

[86] Buratowski S. (1994). Cell..

[87] Alexiadis V., Waldmann T., Andersen J., Mann M., Knippers R., Gruss C. (2000). Genes Dev..

[88] Ganz M., Vogel C., Czada C., Jorke V., Gwosch E.C., Kleiner R., Mach A.P., Zanacchi F.C., Diaspro A., Kappes F. (2019). PLoS One..

[89] Kappes F., Fahrer J., Khodadoust M.S., Tabbert A., Strasser C., Mor-Vaknin N., Moreno-Villanueva M., Bürkle A., Markovitz D.M., Ferrando-May E. (2008). Mol. Cell. Biol..

[90] Soares L.M., Zanier K., Mackereth C., Sattler M., Valcarcel J. (2006). Science..

[91] Campillos M., Garcia M.A., Valdivieso F., Vazquez J. (2003). Nucleic Acids Research.

[92] Gamble M.J., Fisher R.P. (2007). Nat. Struct. Mol. Biol..

[93] Sanden C., Jarvstrat L., Lennartsson A., Brattas P.L., Nilsson B., Gullberg U. (2014). Mol. Cancer..

[94] Wise-Draper T.M., Allen H.V., Jones E.E., Habash K.B., Matsuo H., Wells S.I. (2006). Mol. Cell. Biol..

[95] Broxmeyer H.E., Mor-Vaknin N., Kappes F., Legendre M., Saha A.K., Ou X., O’Leary H., Capitano M., Cooper S., Markovitz D.M. (2013). Stem Cells..

[96] Mor-Vaknin N., Rivas M., Legendre M., Mohan S., Yuanfan Y., Mau T., Johnson A., Huang B., Zhao L., Kimura Y. (2018). Arthritis Rheumatol..

[97] Fahrer J., Popp O., Malanga M., Beneke S., Markovitz D.M., Ferrando-May E., Bürkle A., Kappes F. (2010). Biochemistry..

[98] Malanga M., Atorino L., Tramontano F., Farina B., Quesada P. (1998). Biochim. Biophys. Acta..

[99] Althaus F.R. (1992). J. Cell. Sci..

[100] Thomas C., Ji Y., Wu C., Datz H., Boyle C., MacLeod B., Patel S., Ampofo M., Currie M., Harbin J. (2019). Proc. Natl. Acad. Sci. USA..

[101] Sultanov D.C., Gerasimova N.S., Kudryashova K.S., Maluchenko N.V., Kotova E.Y., Langelier M.F., Pascal J.M., Kirpichnikov M.P., Feofanov A.V., Studitsky V.M. (2017). AIMS Genet..

[102] Maluchenko N.V., Sultanov D.S., Kotova E.Y., Kirpichnikov M.P., Studitsky V.M., Feofanov A.V. (2019). Dokl. Biochem. Biophys..

[103] Maluchenko N.V., Kulaeva O.I., Kotova E., Chupyrkina A.A., Nikitin D.V., Kirpichnikov M.P., Studitsky V.M. (2015). Mol. Biol. (Moskow)..

[104] Popp O., Veith S., Fahrer J., Bohr V.A., Burkle A., Mangerich A. (2013). ACS Chem. Biol..

[105] Lebel M., Monnat R.J. Jr. (2018). Ageing Res. Rev..

[106] Orlovetskie N., Serruya R., Abboud-Jarrous G., Jarrous N. (2017). Biochim. Biophys. Acta Rev. Cancer..

[107] D’Annessa I., Coletta A., Desideri A. (2014). Biopolymers..

[108] Miwa M., Saikawa N., Yamaizumi Z., Nishimura S., Sugimura T. (1979). Proc. Natl. Acad. Sci. USA..

[109] Chen Q., Kassab M.A., Dantzer F., Yu X. (2018). Nat. Commun..

[110] Alemasova E.E., Lavrik O.I. (2019). Nucleic Acids Research.

[111] Sukhanova M.V., Abrakhi S., Joshi V., Pastre D., Kutuzov M.M., Anarbaev R.O., Curmi P.A., Hamon L., Lavrik O.I. (2016). Nucleic Acids Research.

[112] Mehrotra P.V., Ahel D., Ryan D.P., Weston R., Wiechens N., Kraehenbuehl R., Hughes T.O., Ahel I. (2011). Molecular Cell.

[113] Riccio A.A., Cingolani G., Pascal J.M. (2016). Nucleic Acids Research.

[114] Aravind L., Koonin E.V. (2000). Trends Biochem. Sci..

[115] Ali A.A., Timinszky G., Arribas-Bosacoma R., Kozlowski M., Hassa P.O., Hassler M., Ladurner A.G., Pearl L.H., Oliver A.W. (2012). Nat. Struct. Mol. Biol..

[116] Haince J.F., McDonald D., Rodrigue A., Dery U., Masson J.Y., Hendzel M.J., Poirier G.G. (2008). J. Biol. Chem..

[117] Langelier M.F., Planck J.L., Roy S., Pascal J.M. (2012). Science..

[118] Mortusewicz O., Ame J.C., Schreiber V., Leonhardt H. (2007). Nucleic Acids Research.

[119] Zhang H., Ji X., Li P., Liu C., Lou J., Wang Z., Wen W., Xiao Y., Zhang M., Zhu X. (2020). Sci. China Life Sci..

[120] Gao X.M., Zhang Z.H. (2020). Yi Chuan..

[121] Banani S.F., Rice A.M., Peeples W.B., Lin Y., Jain S., Parker R., Rosen M.K. (2016). Cell..

[122] Li P., Banjade S., Cheng H.C., Kim S., Chen B., Guo L., Llaguno M., Hollingsworth J.V., King D.S., Banani S.F. (2012). Nature.

[123] Nakashima K.K., Vibhute M.A., Spruijt E. (2019). Front Mol. Biosci..

[124] Yamamoto T., Yamazaki T., Hirose T. (2020). Soft Matter..

[125] Kohata K., Miyoshi D. (2020). Biophys. Rev..

[126] Schultheisz H.L., Szymczyna B.R., Williamson J.R. (2009). J. Am. Chem. Soc..

[127] Singatulina A.S., Hamon L., Sukhanova M.V., Desforges B., Joshi V., Bouhss A., Lavrik O.I., Pastré D. (2019). Cell Rep..

[128] McGurk L., Gomes E., Guo L., Mojsilovic-Petrovic J., Tran V., Kalb R.G., Shorter J., Bonini N.M. (2018). Molecular Cell.

[129] Leung A.K. (2014). J. Cell. Biol..

[130] Mastrocola A.S., Kim S.H., Trinh A.T., Rodenkirch L.A., Tibbetts R.S. (2013). J. Biol. Chem..

[131] Balagopal V., Parker R. (2009). Curr. Opin. Cell Biol..

[132] Boehning M., Dugast-Darzacq C., Rankovic M., Hansen A.S., Yu T., Marie-Nelly H., McSwiggen D.T., Kokic G., Dailey G.M., Cramer P. (2018). Nat. Struct. Mol. Biol..

[133] Lu H., Liu R., Zhou Q. (2019). Transcription..

[134] Portz B., Shorter J. (2020). Trends Biochem. Sci..

[135] Krishnakumar R., Kraus W.L. (2010). Molecular Cell.

[136] Nalabothula N., Al-jumaily T., Eteleeb A.M., Flight R.M., Xiaorong S., Moseley H., Rouchka E.C., Fondufe-Mittendorf Y.N. (2015). PLoS One..

